# Genetic variation in *Interleukin-32* influence the immune response against New World *Leishmania* species and susceptibility to American Tegumentary Leishmaniasis

**DOI:** 10.1371/journal.pntd.0008029

**Published:** 2020-02-05

**Authors:** Jéssica Cristina dos Santos, Valéria Bernadete Leite Quixabeira, Muriel Vilela Teodoro Silva, Michelle S. M. A. Damen, Kiki Schraa, Martin Jaeger, Marije Oosting, Samuel T. Keating, Miriam Leandro Dorta, Sebastião Alves Pinto, Fernanda Bugalho Duarte, Ledice Inácia de Araújo Pereira, Mihai G. Netea, Fátima Ribeiro-Dias, Leo A. B. Joosten

**Affiliations:** 1 Radboud Institute for Molecular Sciences (RILMS), Department of Internal Medicine and Radboud Center of Infectious Diseases (RCI), Radboud University Medical Center, Nijmegen, The Netherlands; 2 Laboratório de Imunidade Natural (LIN), Instituto de Patologia Tropical e Saúde Pública, Universidade Federal de Goiás, Goiânia, Goiás, Brazil; 3 Instituto Goiano de Oncologia e Hematologia (INGOH), Goiânia, Goiás, Brazil; 4 Division of Immunobiology, Cincinnati Children’s Hospital Medical Center, Cincinnati, Ohio, United States of America; 5 Faculdade de Medicina, Universidade Federal de Goiás, Goiânia, Goiás, Brazil; 6 Hospital Unique, Goiânia, Goiás, Brazil; 7 Hospital de Doenças Tropicais Anuar Auad, Goiânia, Goiás, Brazil; 8 Department for Genomics & Immunoregulation, Life and Medical Sciences Institute (LIMES), University of Bonn, Bonn, Germany; Universiteit Antwerpen, BELGIUM

## Abstract

Interleukin-32 is a novel inflammatory mediator that has been described to be important in the immunopathogenesis and control of infections caused by *Leishmania* parasites. By performing experiments with primary human cells *in vitro*, we demonstrate that the expression of IL-32 isoforms is dependent on the time exposed to *L*. *amazonensis* and *L*. *braziliensis* antigens. Moreover, for the first time we show the functional consequences of three different genetic variations in the *IL32* (rs4786370, rs4349147, rs1555001) modulating IL-32γ expression, influencing innate and adaptive cytokine production after *Leishmania* exposure. Using a Brazilian cohort of 107 American Tegumentary Leishmaniasis patients and a control cohort of 245 healthy individuals, the *IL32* rs4786370 genetic variant was associated with protection against ATL, whereas the *IL32* rs4349147 was associated with susceptibility to the development of localized cutaneous and mucosal leishmaniasis. These novel insights may help improve therapeutic strategies and lead to benefits for patients suffering from *Leishmania* infections.

## Author summary

In this study, we described how IL-32 isoforms are crucial to host defense against new world *Leishmania* species infections. Furthermore, by accessing the genotype frequency of genetic variations in *IL32* in a cohort of Brazilian patients with American Tegumentary Leishmaniasis (ATL) and controls, we have obtained indications that IL-32 is associated with disease susceptibility and the development of different clinical manifestations. Thus, this study provides us an extra evidence that the isoforms of IL-32 shape the immune response favoring the development of different cytokines produced by peripheral blood mononuclear cells that might contribute to skin/mucosal inflammation and host defense.

## Introduction

American Tegumentary Leishmaniasis (ATL) is a chronic inflammatory disease caused by protozoa of the genus *Leishmania*. In Brazil, it is caused by species that belong to *Leishmania* (*Viannia*) and *Leishmania* (*Leishmania*) subgenus, which can lead to different clinical manifestations. The localized cutaneous leishmaniasis (LCL) is one of the most frequent and mild forms of disease, which can cure spontaneously in a minority of the leishmaniasis infected patients. However, the other forms of ATL including mucosal leishmaniasis (ML), disseminated leishmaniasis (DL), and diffuse cutaneous lesions (DCL) are more difficult to treat, and relapses are highly frequent [1,2].

Importantly, the clinical manifestation of leishmaniasis is dependent upon both the parasite species and the host’s immune response. For example, *Leishmania* (*V*.*) braziliensis* and *Leishmania* (*L*.) *amazonensis* primarily cause LCL, which is characterized by one or few ulcers in the skin with elevated borders, appearing mainly in exposed areas, like upper limbs, lower limbs and face. Among the severe cases, *L*. *braziliensis* causes ML affecting primarily the nasopharyngeal and oral mucosal epithelial barriers, often leading to ulceration and septum perforation. DL is characterized by more than ten papular and ulcerated lesions spread on at least two non-contiguous areas of the patient’s body [3,4]. DCL is a rare but severe form of the disease caused by *L*. *amazonensis* characterized by the presence of non-ulcerative disseminated nodules that often affects face, limbs and trunk of patients [5,6].

Upon inoculation of *Leishmania* spp. by a sand fly into the host skin, cellular and molecular events can influence the development of the disease. Innate immune cells are important during early stages of infection. Dendritic cells (DC), natural killer (NK) cells, neutrophils, and macrophages are fundamental for the host response to the parasite [7,8]. Additionally, pattern recognition receptors (PRR) are critical for *Leishmania* parasite recognition by host immune cells. The major pathogen associated molecular pattern (PAMP) in *Leishmania* is lipophosphoglycan (LPG) [9]. In case of *L*. *braziliensis* [10] and *L*. *amazonensis* [11], their LPG can be recognized by Toll-like receptor (TLR) 2 and TLR4, respectively. Toll-like receptors (TLRs), C-type lectin receptors (CLR) dectin-1 and mannose receptor (MR), complement receptors CR1 and CR3, Fc receptors and NOD-like receptor (NLR) have been reported as important for *Leishmania*-host interface [12–14].

In addition to its well-described function in parasite control, IFNγ has been associated with immunopathology in ATL. Furthermore, TNFα, IL-1β, IL-17 and CD8^+^ T lymphocytes have also been implicated in promoting pathology in leishmaniasis. [15–18]. Contrary to inflammatory cytokines, IL-10 can regulate some of these immunopathologic responses [19,20]. Moreover, other members of the IL-10 family have been identified as key players in the wound healing process [21,22]. IL-22 is one of the members of this family, which has been pointed as an important factor to limit *Leishmania*-induced immunopathology [23].

IL-32 is another cytokine that has been described to be involved in the immunopathogenesis of inflammatory diseases and control of intracellular pathogens. IL-32 is mainly produced by immune cells, but it is additionally expressed in non-immune cells including epithelial and endothelial cells [24–28]. IL-32 mRNA can be processed by alternative splicing, generating several different isoforms. The isoforms are generated from the pre-mRNA for IL-32γ, which in respect to inflammation is the most active isoform. In the context of human diseases, the most studied isoforms are IL-32α, IL-32β and IL-32γ [29,30]. On the functional level, IL-32γ amplifies inflammatory signaling induced by TNFα and synergizes with PRRs for the induction of IL-1β and IL-6 [31,32]. Through these mechanisms, IL-32 drives innate immune cells to generate Th1 and Th17 cellular responses [33]. From a molecular perspective, IL-32 can inhibit proinflammatory cytokine mRNA decay influencing the activation of transcription factors [26]. Furthermore, IL-32γ inhibits Hepatitis B virus activities at the transcriptional level through a non-cytokine function in hepatocytes [34].

We previously described the expression of IL-32 in epithelial, endothelial and mononuclear cells in lesions from patients with LCL and ML [35]. In fact, IL-32 has also been implicated as a key player in controlling parasite load in infections caused by *L*. *braziliensis* or *L*. *amazonensis* in both the IL-32γ-transgenic mouse model and human cells [36,37]. To date, it is not precisely known how IL-32 isoforms contribute to host defense against *Leishmania* infections. In the current study, we confirmed that *IL32* mRNA and protein are expressed in human PBMCs stimulated with *L*. *amazonensis* or *L*. *braziliensis*. We further evaluated the functional consequences of three different genetic variations in *IL32* (rs4786370, rs4349147, rs1555001) for innate and adaptive cytokine production. Furthermore, by analyzing a cohort of 107 ATL patients and 245 controls, we evaluated whether these genetic variations in *IL32* influence the susceptibility to disease and the clinical outcome. Finally, the expression of important inflammatory mediators in lesion fragments from ATL patients was correlated with mRNA levels of IL-32 isoforms.

## Materials and methods

### Patients, controls and tissues samples

The Brazilian cohort of ATL patients: to access genetic variations, ATL patients (n = 107) were asked to provide blood. All patients were diagnosed with cutaneous (n = 68), mucosal (n = 38) or diffuse (n = 1) ATL in Annuar Auad Tropical Disease Hospital, in Goiânia, Goiás, West Central region of Brazil. To be included in the study, besides a leishmaniasis lesion, patients had at least one exam indicating the presence of parasites were included. Among the technical procedures used for the diagnosis, polymerase chain reaction (PCR) for *Leishmania* genus, histopathological exam with immunohistochemistry for amastigotes forms (IHC), indirect immunofluorescence (IFI), and Montenegro’s skin test (MST) were used (Table 1) [38]. For cytokine expression evaluation, fragments of cutaneous and mucosal lesions of 56 ATL patients were obtained. All the fragments were tested for parasite DNA using a polymerase chain reaction (PCR-RFLP) described by [39], and presented positive result for *Leishmania Viannia* subgenus. For gene expression evaluation, one lesion fragment was stored in TRIzol reagent (Invitrogen, USA). For the controls (n = 245), blood from healthy individuals was obtained at the blood bank of the Goiano Institute of Oncology and Hematology (INGOH), in Goiânia, Goiás. All procedures were approved by Ethics Committee of Hospital das Clínicas and Hospital Anuar Auad (CAE 47895015.0.0000.5078) and of Federal University of Goiás (n. 164/08), Goiânia, Goiás, Brazil and informed consent was signed by all patients and controls. An additional study was performed using venous blood obtained from 3 Crohn’s disease patients bearing the NOD2 3020insC mutation, blood of 6 healthy individuals was used as control. All human subjects of the study were adults.

**Table 1:** 

Characteristics of ATL patients^#^			
	LCL*	ML^$^	DL^&^
Male	53/68	26/38	-
Female	15/68	(12/38)	1 ± 0
Age (Years)	43.7 ± 14.2	56.4 ± 15.0	41 ± 0
Number of lesions	2.0 ± 1.7	-	20 ± 0
MST (mm)^@^	11.6 ± 6.7	25.2 ± 15.7	-
**Positive diagnostic test results % (positive/total tested)**			
PCR^^^	80.9 (17/21)	100 (8/8)	-
IHC^+^	75.0 (33/44)	62.5 (10/16)	-
IFI^¶^	62.0 (18/29)	68.1 (15/22)	Positive
MST^@^	48.3 (15/31)	73.3 (11/15)	-

^#^One hundred and seven ATL patients who were assisted at Hospital de Doenças Tropicais Anuar Auad (Goiânia—Goiás, West Central, Brazil), 68 with ^*^LCL (Localized Cutaneous Leishmaniasis), 38 with ^$^ML (Mucosal leishmaniasis) and 1 with DL^&^ (Disseminated Leishmaniasis).

^^^PCR: polymerase chain reaction for *Leishmania* genus

^+^IHC: histopathological exam with immunohistochemistry for amastigotes

^@^ MST: Montenegro skin test

^¶^IFI: indirect immunofluorescence; 72 patients were from West Central region of Brazil (including 25 with ML and 1 with DL); 16 from North (including 4 with ML) and 1 from North East; For remaining 18 patients the data was missing.

### *Leishmania* cultures and lysates

*L*. *amazonensis* (IFLA/BR/67/PH8) reference strain and MHOM/BR/2003/IMG *L*. *braziliensis*, a strain from *Leishbank* IPTSP/UFG, were used. Promastigote forms were cultured in Grace’s insect medium (Gibco, Life Technologies, USA) supplemented with 20% of heat-inactivated fetal bovine serum (FBS, Gibco), 100 U/mL of penicillin/streptomycin (Sigma-Aldrich), at 26°C. Stationary-phase parasites were obtained on the 6th day of growth and washed three times with phosphate-buffered saline (PBS; 1000 x g, 10 min 10°C). Parasite lysates were obtained by 5 freeze-thaw cycles of promastigotes in the presence of protease inhibitors (Protease inhibitor cocktail, Sigma-Aldrich), in liquid nitrogen followed by thawing in a water bath at 37°C. Protein quantification was performed using the Pierce BCA protein assay kit (ThermoFisher Scientific, USA).

### Isolation of peripheral blood mononuclear cells (PBMC) from healthy controls and treatments

Peripheral blood mononuclear cells (PBMCs) were isolated from buffy coats obtained after informed consent of healthy volunteers (Sanquin BloodBank, Nijmegen) and from EDTA tubes of homozygous individuals for the *NOD2* 3020insC mutation, presenting Crohn’s disease. PBMCs were obtained by Ficoll-Paque density gradient cell isolation (GE healthcare, UK), as previously described [12,40]. Cells were resuspended in RPMI 1640 culture medium (Roswell Park Memorial Institute medium; Invitrogen, USA) supplemented with 50 μg/mL gentamicin, 2 mM glutamax (Gibco), and 1 mM pyruvate (Gibco) and quantified. In brief, PBMC stimulations with lysates of both *L*. *amazonensis* or *L*. *braziliensis* (50 μg/mL) were performed with 5 x 10^5^ cells/well in round-bottom 96-wells plates for either 24 h or 7 days in the presence of culture medium with 10% human pool serum at 37°C and 5% CO_2._ In addition, PBMCs were stimulated during 24 h with LPG (10 μg/mL) from *L*. *amazonensis* or *L*. *braziliensis*. In some experiments, PBMCs were pre-incubated for 1 h, at 37°C, and 5% CO_2_ with either *Bartonella quintana LPS* (5 μg/mL) [40] for TLR4 blocking or with laminarin (100 μg/mL; Sigma-Aldrich) as antagonist of Dectin-1. After the stimulation period, supernatants and cells were stored in either 200 μL of TRIzol (Invitrogen) at -80°C until RNA extraction or in 100 μL of 0.5% TritonX-100 (Sigma-Aldrich) for intracellular IL-32 measurement.

### Genetic analysis in the 200FG cohort

Genetic variation was assessed in the *IL-32* gene in healthy individuals of Western European descent from the 200FG cohort [41], (http://www.humanfunctionalgenomics.org/). The volunteers were between 23 and 73 years old and consisted of 77% males and 23% females. PBMCs were isolated as described above. After 24 h and 7 days, supernatants were collected and cytokines were measured by enzyme-linked immunosorbent assay (ELISA) as described below.

### Transcriptional analysis

Previously published microarray data of normal skin samples (n = 10), early (n = 8) and late (n = 17) LCL lesion samples were obtained form the publicly available National Center for Biotechnology Information (NCBI) Gene Expression Omnibus (GEO) database (accession number GSE55664) [42]. The expression of selected genes in normal skin versus *L*. *braziliensis* lesions were represented in a heatmap presented as dendograms shown as a tree, representing the distance between variables. Unsupervised hierarchical clustering of selected genes was performed using the R package hclust with Spearman correlation and presented using the R package ‘corrplot’.

### Quantitative PCR

RNA was isolated from PBMCs and ATL patient’s lesions stored in TRIzol as previously described [43]. cDNA was made using the iScript kit (Bio-Rad). Primer pairs for the *IL32* isoforms are described elsewhere [36]. *IL1B*, *IL1A*, *IL22*, *IL-17 and IFNG* primers sequences were designed specifically for each transcript using Primer Express 3.0 software (DeNovo Software, Glendale, CA, USA). Primers were produced by Sigma-Aldrich. Diluted cDNA was used for qPCR that was done by using the StepOnePlus qPCR system (Applied Biosystems, Foster City, CA, USA) with SYBR Green Mastermix (Applied Biosystems). Fold change and relative expression were calculated with the 2^-ddCT and 2^-dCT methods, respectively and normalized against the housekeeping gene *B2M*.

### Enzyme-linked immunosorbent assay (ELISA)

Cytokine production was determined in 24 h supernatants using commercial ELISA kits (R&D Systems) for human TNFα, IL-6 and IL-1β. Supernatants of the 7 days stimulation were used to measure IFNγ, IL-17A and IL-22. IL-32 was determined intracellularly in Triton X-100-cell lysates after 24 h and 7 days using commercial ELISA kit (R&D Systems).

### Isolation of genomic DNA and genetic assessment of *IL32* variations in ATL patients and controls

DNA was isolated from venous blood of ATL patients and healthy controls using the illustra blood genomicPrep Mini Spin Kit (GE Healthcare, Little Chalfont, UK), according to the manufacturer’s protocol. SNPs in *IL32* were selected based on previously published functional effects on protein function and/or gene expression and characteristics are described in detail in Supplementary Table 1 [44,45]. Genotyping of ATL patients and controls was performed by TaqMan SNP assays (S1 Table) (Applied Biosystems), according to the manufacturer’s protocol on the StepOnePlus qPCR system (Applied Biosystems). Quality control was performed by the incorporation of positive and negative controls and duplication of random samples across different plates.

### Statistical analysis

All statistical analyses and graphs of cytokine concentrations and mRNA expression in the *in vitro* PBMC stimulation assays were performed and created using GraphPad Prism 6.00 software (GraphPad Software, San Diego, CA, USA). Data are shown as mean ± SEM. Results were stratified by genotype, and differences in cytokine production between genotypes were analyzed by Mann–Whitney U test, otherwise in each figure, the applied statistical test is indicated in the figure legend. The difference in genotype frequencies between the patients and the control group was analyzed in a dominant and a recessive model using logistic regression. No correction for multiple testing was performed. The effect of the genotypes on ATL susceptibility was estimated by calculating odds ratios (ORs) and their 95% confidence intervals using the same statistical methods. We also performed Fisher test analysis to determine whether clinical manifestations of ATL were associated with one of the *IL32* genotypes. Analyses were conducted using the statistical computing evrionment, R (v3.5.0). Overall, statistical tests *P*-values of < 0.05 were considered to be statistically significant.

## Results

### Differential IL-32 isoform expression is dependent on duration of exposure to *Leishmania* antigens

**Fig 1:**
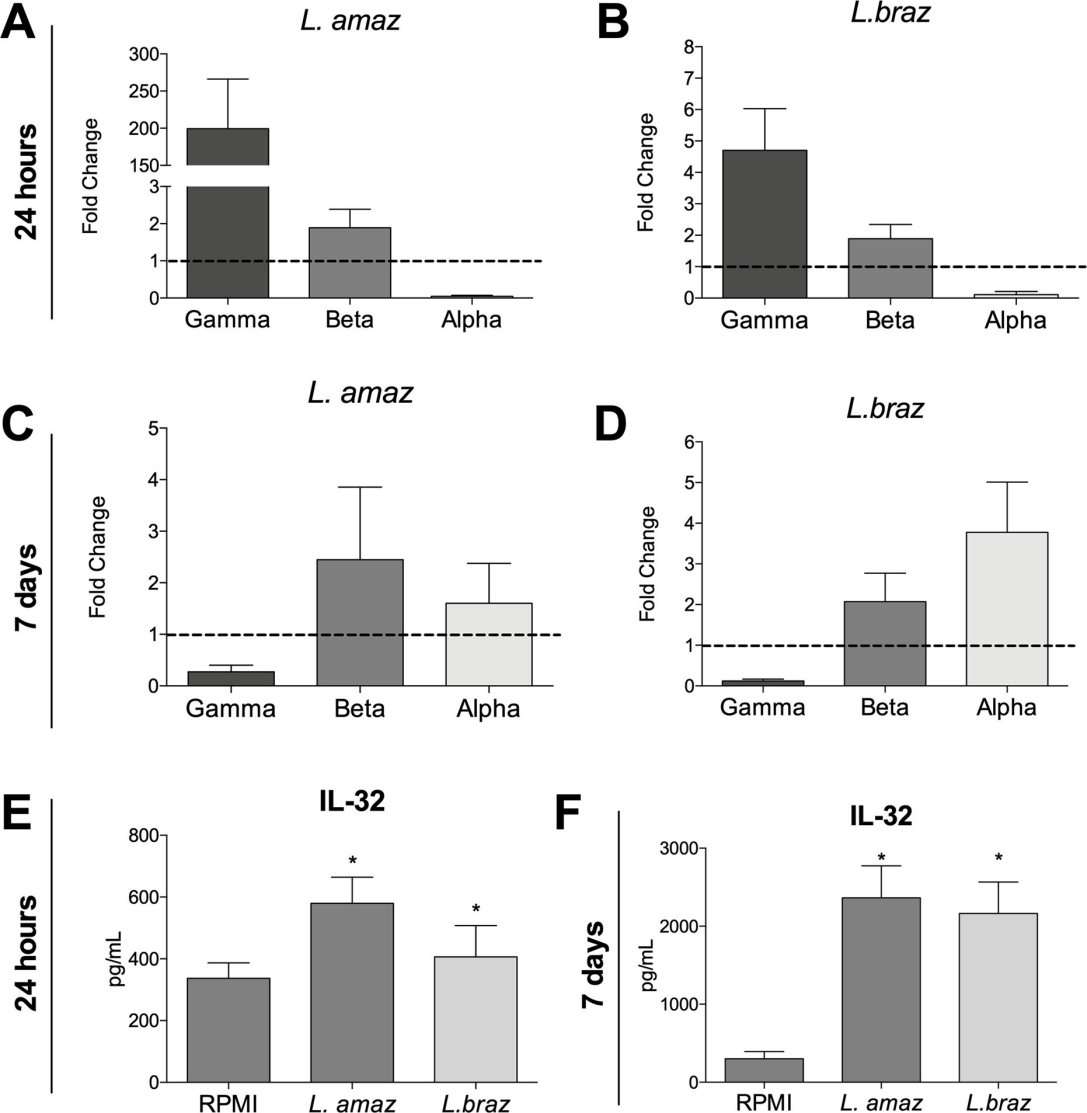
*IL-32* isoform gene expression as well as intracellular IL-32 protein were measured in PBMCs stimulated with lysates (50 μg/ mL) of *L*. *amazonenis* and *L*. *braziliensis* for (A-B-E) 24 hours and (C-D-F) 7 days. The data shown are the mean ± SEM of fold change in *IL32* expression induced by *Leishmania* spp. compared to RPMI control (dotted line). Results are compiled from at least two independent experiments. The data shown are the mean ± SEM, n = 6. *p < 0.05 (RPMI vs *Leishmania* spp., Wilcoxon test).

To investigate whether *Leishmania* species can induce different isoforms of *IL32* in PBMCs from healthy individuals in a time-dependent manner, we evaluated *IL32* mRNA expression. Both *L*. *amazonensis* and *L*. *braziliensis* induced IL-32γ and IL-32β expression in PBMCs after incubation for 24 h (Fig 1A and 1B). On the other hand, after 7 days of *Leishmania*-antigen stimulation, the IL-32β and IL-32α were the main isoforms expressed (Fig 1C and 1D). The production of intracellular IL-32 protein by PBMCs upon exposure to antigens from both *Leishmania* species was enhanced compared to unstimulated cells at both time points (Fig 1E and 1F). However, we observed a 5-fold increase in intracellular IL-32 between 7 days (Fig 1F) and 24 h of incubation (Fig 1E).

### Early induction of IL-32γ is mediated by NOD2, TLR4 and Dectin-1

In order to evaluate which are the receptors involved in early IL-32 induction by *Leishmania* spp. in PBMCs, we further assessed whether genetic variations in PRRs could affect the expression of IL-32γ expression. Supplemental Fig 1A (S1A Fig) shows a reduction of IL-32γ mRNA expression in PBMCs isolated from individuals carrying a frame shift mutation in the NOD2 receptor (rs2066847- leu1007insC), when compared to individuals carrying no mutation (Wt) after 24 h of exposure to *L*. *amazonensis* antigens. Exploring TLR2 and TLR4, we observed that a missense SNP in *TLR4* (rs4986791), but not in *TLR2* (rs5743708), modulates IL-32γ mRNA expression upon *L*. *amazonensis* exposure. In addition, we investigated the role of C-type lectin receptors, more specifically Dectin-1 (*CLEC7A* rs16910526 SNP) and Mannose receptor (*CD206* rs1926736 SNP) role in IL-32γ induction. S1B Fig (S1B Fig) shows that a nonsense SNP in *CLEC7A* might influence IL-32γ mRNA expression, since one individual carrying the CC genotype presented lower IL-32γ mRNA expression compared to individuals bearing AA and AC genotypes. This effect was seen for both species of *Leishmania*. No effect on IL-32γ mRNA expression was observed for individuals carrying different genotypes for a missense SNP in *CD206* (rs1926736).

**Fig 2:**
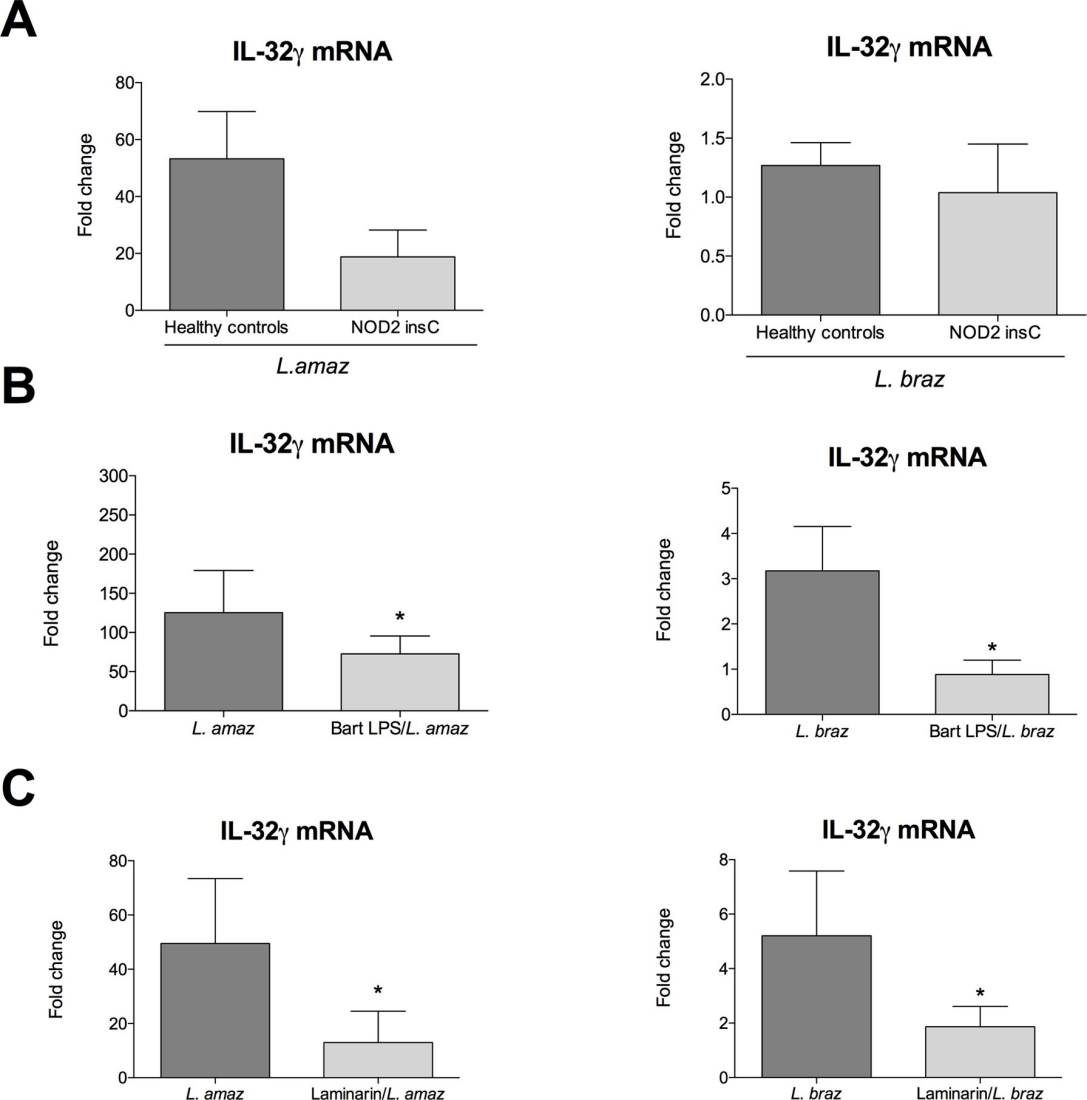
IL-32γ gene expression was measured in PBMCs from healthy controls carrying no mutation in *NOD2* (n = 6) and from individuals carrying a mutation in *NOD2* receptor (3020insC) (n = 3) after 24 hours exposure with lysates (50 μg/mL) of *L*. *amazonenis* and *L*. *braziliensis* (A-B). IL-32γ gene expression measured in PBMCs from healthy individuals (n = 6) pre-incubated 1 hour in the presence of *Bartonella quintana LPS* (Bart LPS—5 μg/mL) and with laminarin (100 μg/mL) followed by stimulation with lysates of *L*. *amazonenis* and *L*. *braziliensis* (B-C). Results are compiled from at least two independent experiments. The data shown are the mean ± SEM, n = 6. *p < 0.05 (*Leishmania* spp. vs *Leishmania* spp. in the presence of Bart LPS or laminarin, Wilcoxon test).

The influence of NOD2, TLR4 and Dectin-1 signaling on IL-32γ induction upon *Leishmania* spp. exposure was further validated using biological assays. PBMCs isolated from patients carrying a frameshift mutation in NOD2 expressed lower levels of IL-32γ mRNA than healthy individuals after *L*. *amazonensis* stimulation. There were no differences in *L*. *braziliensis*-stimulated cells (Fig 2A). In addition, when TLR4 was blocked with *Bartonella quintana* LPS (TLR4 antagonist [40] there was a significant reduction in *Leishmania* spp.-induced IL-32γ mRNA expression (Fig 2B). Next, using laminarin as a Dectin-1 antagonist, we observed a significant reduction in IL-32γ mRNA expression after exposure to both species of *Leishmania*. (Fig 2C).

### Genetic variations in *IL-32* gene influence cytokine production upon *Leishmania* exposure

**Fig 3:**
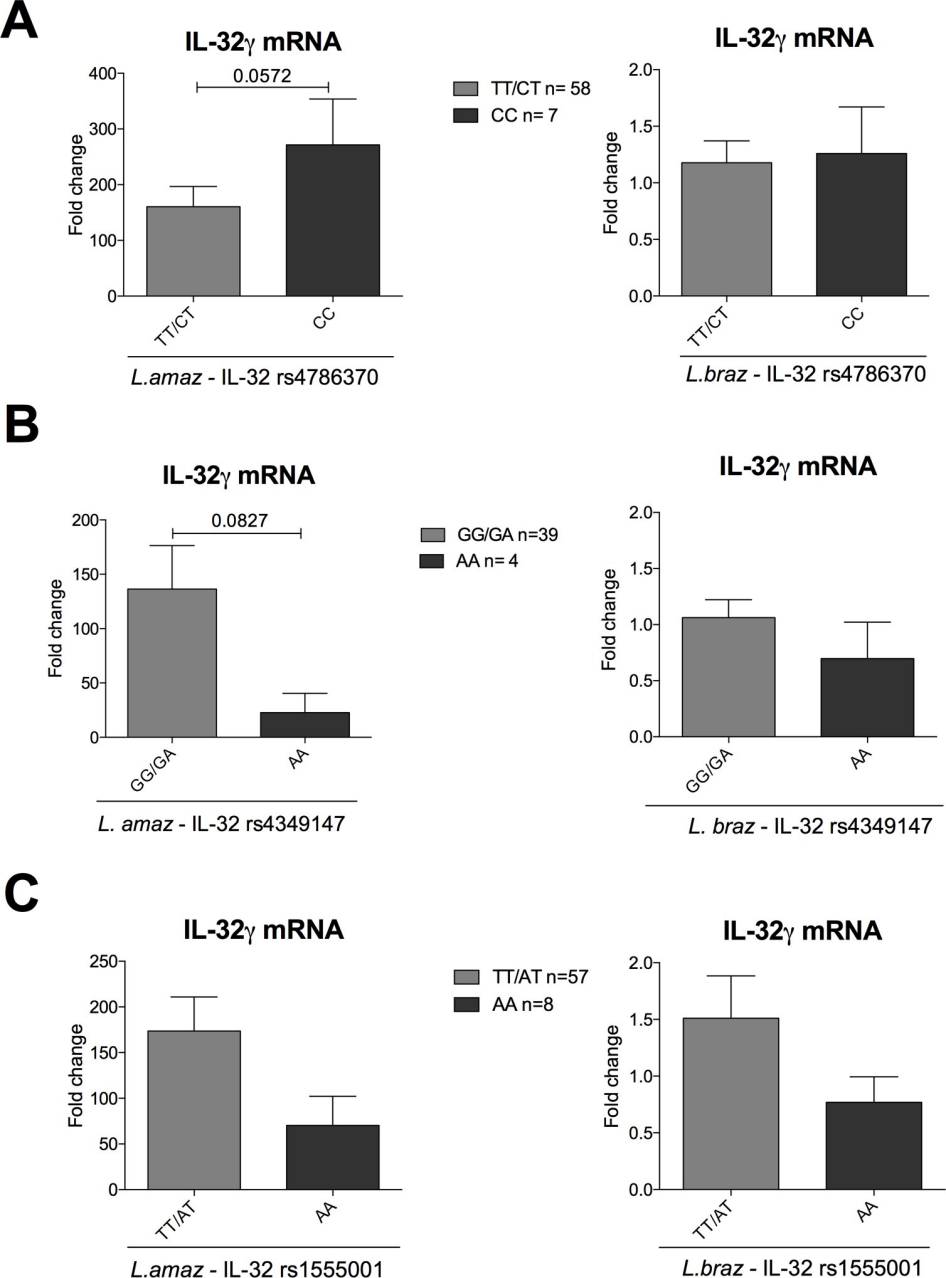
PBMCs from healthy donors from the 200FG cohort were stimulated with lysates (50 μg/mL) of *L*. *amazonenis* and *L*. *braziliensis* for 24 hours. The individuals were stratified according with different genotypes of (A) *IL-32* rs4786370 (TT/CT n = 58; CC n = 7), (B) rs4349147 (GG/GA n = 39; AA n = 4) and (C) rs1555001 (TT/AT n = 57; AA n = 8) and IL-32γ gene expression was determined. Results are compiled from at least two independent experiments. The data shown are the mean ± SEM of fold change in IL-32γ expression induced by *Leishmania* spp. compared to RPMI control.

To further investigate the relevance of IL-32 for *Leishmania*-induced cytokine production, we used functional genomics searching for SNPs within *IL32* that alter gene expression. Therefore, we assessed the response of PBMCs isolated from 200 healthy volunteers (200FG cohort) stimulated with *L*. *amazonensis* or *L*. *braziliensis*. Fig 3A showed that a CC genotype of the promoter SNP (rs4786370) trends to have higher mRNA IL-32γ expression after *L*. *amazonensis* exposure compared to the TT/CT genotypes (p = 0.057). No major differences were observed after *L*. *braziliensis* stimulation. In addition, the effect of a SNP (rs4349147) located in a distal regulatory (enhancer) region of *IL32* was examined. Fig 3B showed that individuals bearing the AA genotypes trends to lower levels of *L*. *amazonensis*-induced IL-32γ mRNA than those bearing GG/GA genotypes (p = 0.08). Again, no differences were observed for *L*. *braziliensis*. In addition, the effect of an intronic SNP (rs1555001) on *IL32* gene expression was investigated. Once again, although no significant differences were observed (p > 0.05), the AA genotypes trends to lower IL-32γ mRNA expression compared to the TT/AT genotypes after exposure to both *Leishmania* species (Fig 3C).

**Fig 4:**
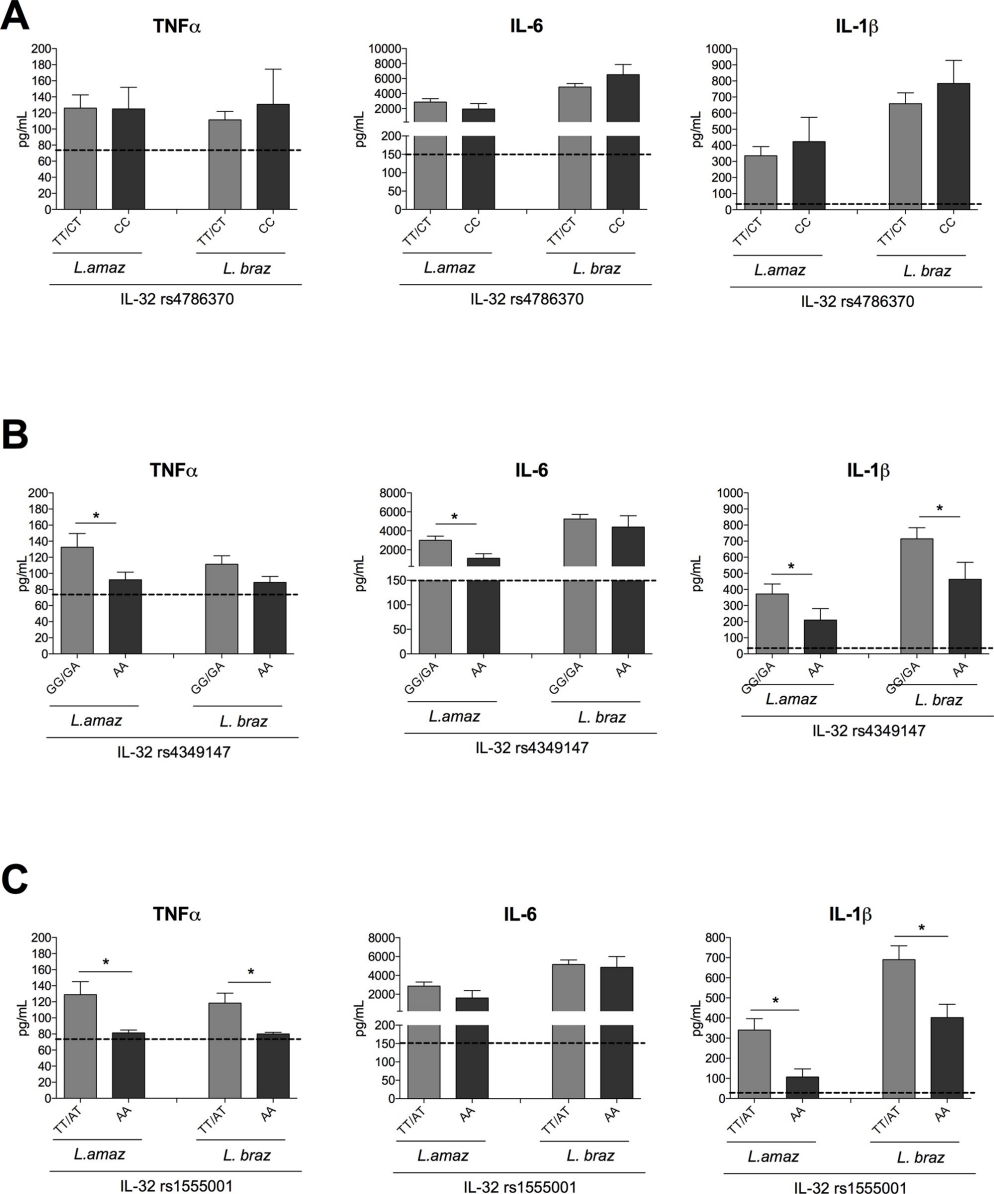
PBMCs from healthy donors from the 200FG cohort were stimulated with lysates (50 μg/mL) of *L*. *amazonenis* and *L*. *braziliensis* for 24 hours. Cytokine levels of TNFα, IL-6 and IL-1β were measured. Cytokine levels were stratified for the different genotypes for (A) *IL-32* rs4786370 (TT/CT n = 139; CC n = 25), (B) rs4349147 (GG/GA n = 140; AA n = 23), (C) rs1555001 (TT/AT n = 146; AA n = 17). Dotted line represents the basal level of cytokine production in the RPMI control. The data shown are the mean ± SEM, *p < 0.05 (TT/CT vs CC; GG/GA vs AA; TT/AT vs AA, respectively, Mann-Whitney U test).

Since genetic variation in the *IL32* gene modulates IL32γ expression, we investigated whether these genetic variations influence both innate and adaptive cytokine production in human PBMCs cultured in the presence of *L*. *amazonensis* or *L*. *braziliensis* antigens. Therefore, we analyzed PBMCs isolated from healthy volunteers of the 200FG cohort with or without those SNPs in the *IL32* gene. The classical innate cytokines TNFα, IL-6 and IL-1β were evaluated after 24 h of PBMC stimulation (early IL-32γ production). No significant differences were observed in any of the examined cytokines within the genotypes of the *IL-32* promoter SNP after both *Leishmania* spp. exposure (Fig 4A). In contrast, a lower TNFα, IL-6 and IL-1β production was observed in the group of individuals with the AA genotypes of the enhancer SNP in *IL-32* than in PBMCs from individuals bearing the GG/GA genotypes after *L*. *amazonensis* stimulation. Moreover, for *L*. *braziliensis* antigen stimulation, a reduction in IL-1β production was observed in cells from individuals carrying the AA genotype of the same SNP compared to the GG/GA genotypes. No significant differences were observed in *L*. *braziliensis*-induced TNFα and IL-6 production (Fig 4B).

**Fig 5:**
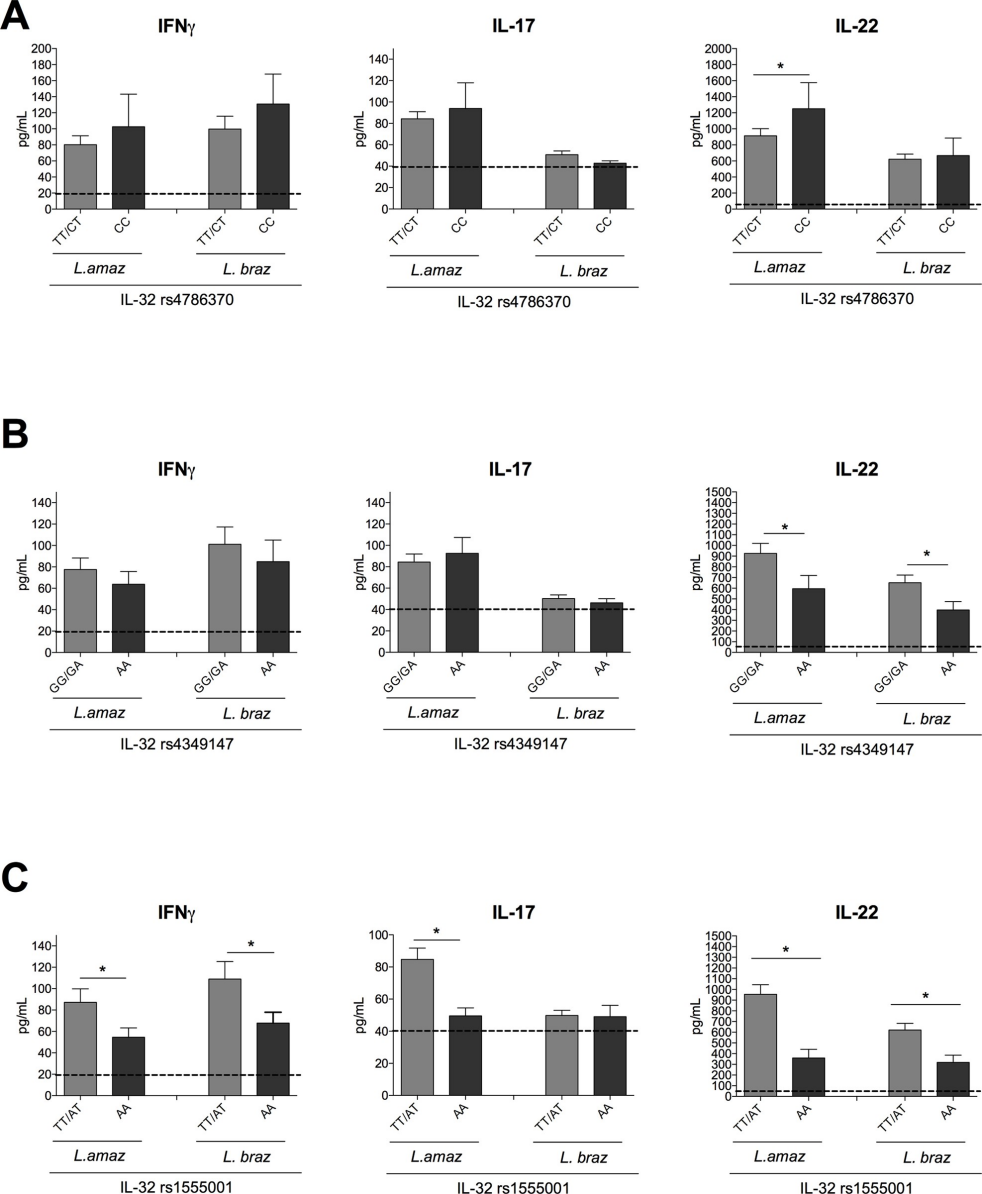
PBMCs from healthy donors from the 200FG cohort were stimulated with lysates (50 μg/ mL) of *L*. *amazonenis* and *L*. *braziliensis* for 7 days. Levels of IFNγ, IL-17 and IL-22 were measured by ELISA. Cytokine levels were stratified for the different genotypes for (A) *IL-32* rs4786370 (TT/CT n = 139; CC n = 25), (B) rs4349147 (GG/GA n = 140; AA n = 23), (C) rs1555001 (TT/AT n = 146; AA n = 17). Dotted line represents the basal level of cytokine production in the RPMI control. The data shown are the mean ± SEM, *p < 0.05 (TT/CT vs CC; GG/GA vs AA; TT/AT vs AA, respectively, Mann-Whitney U test).

For the intronic SNP of *IL32*, significant lower TNFα and IL-1β production was observed in the PBMC cultures of individuals carrying the AA genotype than in those of individuals bearing TT/AT genotypes after stimulation with either species of *Leishmania*. No differences in IL-6 production were observed between the genotypes (Fig 4C). Subsequently, production of the lymphocyte-derived cytokines IFNγ, IL-17 and IL-22 were determined after 7 days of PBMCs cultured with *Leishmania* antigens. Fig 5A shows a higher IL-22 production in the cultures of PBMCs from individuals with CC genotype than those with TT/CT genotypes of the *IL-32* promoter SNP (rs4786370) after *L*. *amazonensis* stimulation. No differences were observed neither in IFNγ nor in IL-17 production when comparing these genotypes. In addition, for the enhancer SNP (rs4349147), IL-22 production was lower in the AA genotype than the GG/GA genotypes after exposure to both *Leishmania* species (Fi 5B). In line with previous SNP data, no differences were observed in IFNγ neither in IL-17 production. Interestingly, for the intronic SNP (rs1555001), the production of IFNγ, IL-17 and IL-22 was lower in the cells with AA genotype, which is associated with low levels of IL-32γ expression after *L*. *amazonensis* stimulation (Fig 5C). Similar results were noted for IFNγ and IL-22 production when PBMCs were stimulated with *L*. *braziliensis*. Strikingly, *L*. *braziliensis* was not capable to induce IL-17 production in the PBMC cultures, in contrast to *L*. *amazonensis* (Fig 5).

### Association of IL-32 genetic variations with susceptibility to *Leishmania* infection

The observation that IL-32 is induced in PBMCs upon stimulation with *Leishmania* spp. and that genetic variations in the *IL32* gene affect IL-32γ expression, and thereby the production of proinflammatory cytokines, led us to the hypothesis that *IL32* SNPs could affect susceptibility to ATL. A Brazilian cohort of 107 ATL patients and a control cohort of 245 healthy individuals were genotyped for *IL32* rs4786370 and rs1555001 genetic variations (Table 2). Different models of genetic variations were tested, including comparison of frequencies of all three genotypes, a dominant model (comparison of frequencies between homozygous wild-type versus heterozygous + homozygous for the allelic variant), and a recessive model (comparison of frequencies between homozygous wild-type + heterozygous versus homozygous for the allelic variant). For the *IL32* rs4786370 variant, the frequency of the CC genotype was significantly higher in controls than in patients (*P* = 0.02). No differences within genotype frequencies were observed for the *IL32* rs1555001 variant between patients and controls (Table 2). Dominant model analyses resulted in no significant different distribution for the *IL32* rs4786370 variant. However, when a recessive model was assumed (CC versus CT + TT), significant difference was observed in the distribution of the homozygous for the variant allele in patients and controls, where patients were less often homozygous (CC) than controls. These results pointed towards an increased frequency of the variant in the cohort of controls, arguing for a decreased risk to develop ATL in the presence of homozygous CC genotype (*P* = 4.00E-03, OR = 0.35 [95% Cl, 0.17–0.72]). No significant associations with susceptibility for both dominant and recessive model analysis were observed in patients and controls carrying both *IL-32* rs1555001 and *IL-32* rs4349147 variations (Table 2).

**Table 2:** 

Difference in genotype frequencies between ATL patients and controls, and the effect of *IL32* genotypes on ATL susceptibility				
Genotype	Patients (%)	Controls (%)	Odds ratio (95% confidence interval)	*P*-value
**rs4786370**				
TT	37 (35%)	82 (34%)	1.00 (Reference)	
CT	60 (56%)	108 (44%)	1.23 (0.72–2.10)	0.44^a^
CC	10 (9%)	55 (22%)	0.40 (0.16–0.91)	0.02^a^
Total	107	245		
***Dominant model***				
TT	37 (35%)	82 (34%)	0.95 (0.58–1.53)	0.83^b^
CT + CC	70 (65%)	163 (66%)		
Total	107	245		
***Recessive model***				
CC	10 (9%)	55 (22%)	0.35 (0.17–0.72)	4.00E-03^b^
CT + TT	97 (91%)	190 (78%)		
Total	107	245		
**rs1555001**				
TT	51 (48%)	132(53%)	1.00 (Reference)	
AT	49 (46%)	96 (39%)	1.31 (0.80–2.17)	0.27^a^
AA	7 (6%)	20 (8%)	0.90 (0.30–2.40)	1^a^
Total	107	248		
***Dominant model***				
TT	51 (48%)	132(53%)	1.24 (0.79–1.96)	0.33^b^
AT + AA	56 (52%)	116 (47%)		
Total	107	248		
***Recessive model***				
AA	7 (6%)	20 (8%)	0.79 (0.32–1.94)	0.62^b^
AT + TT	100 (94%)	228 (92%)		
Total	107	248		
**rs4349147**				
GG	57 (54%)	97(49%)	1.00 (Reference)	
GA	41 (39%)	79 (39%)	1.13 (0.66–1.92)	0.70^a^
AA	7 (7%)	24 (12%)	2.0 (0.77–5.87)	0.14^a^
Total	105	200		
***Dominant model***				
GG	57 (54%)	97(49%)	0.79 (0.49–1.27)	0.33^b^
GA + AA	48 (46%)	103 (51%)		
Total	105	200		
***Recessive model***				
AA	7 (7%)	24 (12%)	0.52 (0.21–1.25)	0.14^b^
GA + GG	98 (93%)	176 (88%)		
Total	105	200		

^**a**^ Fisher test

^**b**^ Logistic regression.

### Association between *IL32* genetic variations and ATL clinical manifestations

To assess whether the *IL32* genetic variation influences clinical manifestations of ATL, patients were stratified according to the clinical forms, including LCL, ML and DL and compared among the different *IL32* genotypes. No statistically significant differences were observed within the frequencies of the genotypes for either *IL-32* rs4786370 and rs1555001variations and the clinical outcome of the patients (Table 3). However, a significant association was observed for the *IL-32* rs4349147 genotypes, with the G allele overrepresented in the group of patients with LCL, whereas the A allele is overrepresented in the group of ML patients (*P* = 0.002). These results suggest that the A allele is associated with susceptibility to develop the more severe form of the disease.

**Table 3:** 

Summary of genotype–phenotype association within the ATL patient group					
**rs4786370**	TT (%)	CT (%)	CC (%)	Total	*P*-value
LCL	23 (61%)	39 (66%)	6 (60%)	68	0.33^a^
ML	15 (39%)	20 (34%)	3 (30%)	38	
DL	0 (0%)	0	1 (10%)	1	
**rs1555001**	TT (%)	AT (%)	AA (%)		
LCL	35 (67%)	29 (60%)	4 (57%)	68	0.71^a^
ML	16 (31%)	19 (40%)	3 (43%)	38	
DL	1 (2%)	0 (0%)	0 (0%)	1	
**rs4349147**	GG (%)	GA (%)	AA (%)		
LCL	35 (61%)	27 (68%)	3 (43%)	65	0.002^a^
ML	21 (37%)	13 (32%)	4 (57%)	38	
DL	1 (2%)	0 (0%)	0 (0%)	1	

^**a**^ Calculated by Fisher test.

### IL-32γ expression is strongly associated with proinflammatory cytokines in ATL skin lesions

**Fig 6:**
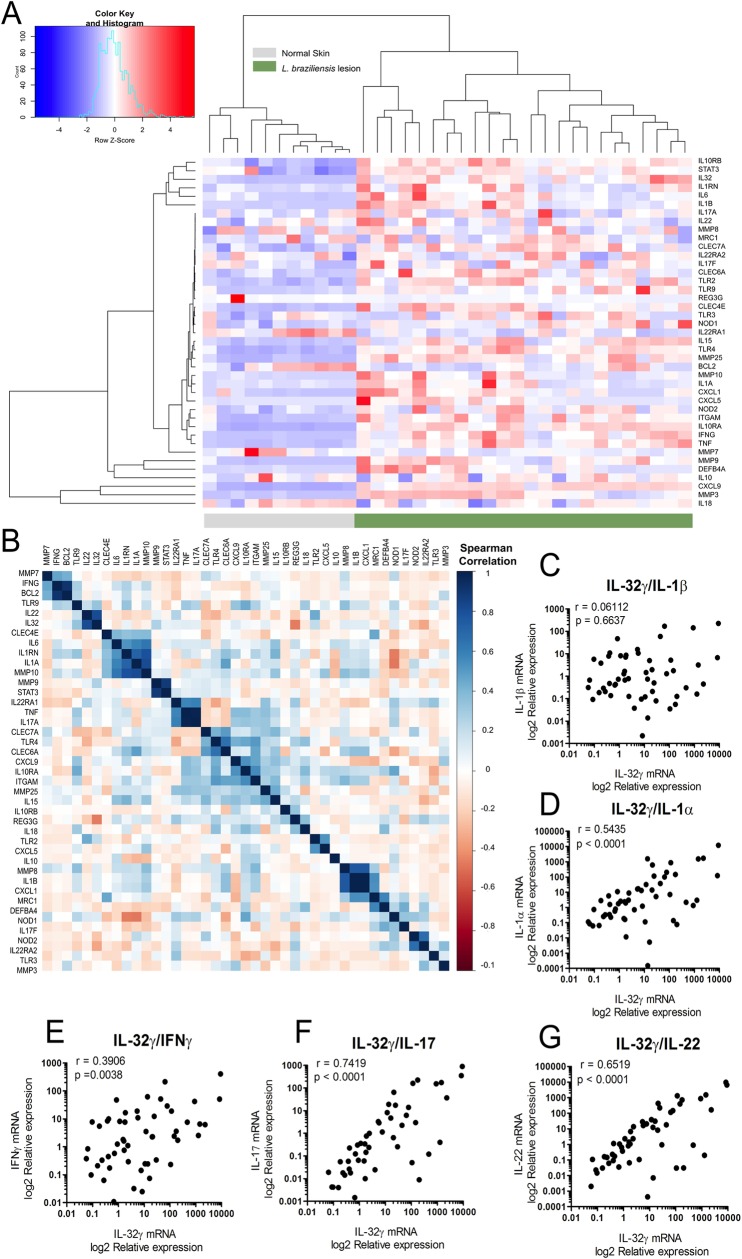
(A) Heatmap showing the expression of selected genes in normal skin versus cutaneous lesions from patients infected with *L*. *braziliensis* described in [42]. Columns represent individual patients and rows represent individual genes, colored to indicate expression levels based on a Z-score. Dendograms are shown as a tree, representing the distance between variables. (B) Correlation plot complementary to A showing the positive (blue) or negative (red) correlations of all genes expressen in cutaneous lesions from patients infected with *L*. *braziliensis* with color intensity reflecting the strength of the correlation. Correlation of IL-32γ with (C) *IL1B*, (D) *IL1A*, (E) *IFNG*, (F) *IL17* and (G) *IL22* gene expression fragments of cutaneous and mucosal lesions of 56 ATL patients determined by qPCR. The data shown are log2 of Relative expression adjusted for beta-2 microglobulin expression (n = 56).

To unravel whether *IL32* expression in skin lesions of LCL patients is associated to genes linked to skin inflammation and host defense, we analyzed the gene expression profile of *L*. *braziliensis* infected skin in comparion to normal skin. The mRNA of the proinflammatory cytokines *IL-32*, *TNF*, *IL1A*, *IL1B*, *IFNG*, *IL-17A* and *IL-22*, the chemokines *CXCL1*, *CXCL5*, *CXCL9*, as well the metalloproteinases *MMP25*, *MMP9*, *MMP10*, *MMP3* and immune receptors *TLR4*, *TLR2*, *NOD1*, *NOD2*, *ITGAM*, *CLEC7A*, *MRC1* were mainly expressed in *L*. *braziliensis* lesions [42] (Fig 6A). Next, we evaluated whether all these genes were correlated to each other in LCL skin lesions (Fig 6B, blue: positive; red: negative correlations). A strong positive correlation was observed between functionally related genes, such as *IL32* and *IL22*, which is adjacent to a another cluster of *CLEC4E*, *IL6*, *IL1RN*, *IL1A*, *MMP10*, *MMP9* genes which are also positively correlated.

Since we demonstrate that genetic variations in *IL32* influence proinflammatory cytokine production in PBMCs stimulated with *Leishmania* spp. (Figs 4 and 5), we were prompted to validate whether IL-32γ expression is associated with innate (*IL1B*, *IL1A*) and adaptive cytokines (*IFNG*, *IL17* and *IL22*) in lesion fragments of our cohort of ATL patients. A significantly positive correlation between IL-32γ expression and *IL1A* (Fig 6D), *IFNG*, and *IL17* (Fig 6E and 6F) was apparent. Of great relevance for host defense, a strong correlation was found between IL-32γ and *IL22* (r^2^ = 0.8930, p < 0.0001) (Fig 6G) expressions in ATL lesions. Remarkably, no significant correlation was observed for IL-32γ and *IL1B* expression (Fig 6C). Moreover, a significant correlation of IL-32β mRNA expression with *IL17* and *IL22* mRNA expression was observed (S2 Fig) whereas IL-32α mRNA expression was significantly correlated with *IL1A* and *IL17* mRNA expression (S3 Fig).

## Discussion

In the present study, we demonstrated that both *L*. *amazonensis* and *L*. *braziliensis* induce IL-32 expression in primary human PBMCs. Of interest, *L*. *amazonensis* is a stronger inducer of both IL-32γ mRNA and protein than *L*. *braziliensis* at least during the first 24 h of PBMC stimulation with *Leishmania* spp. antigens. Intriguingly, when PBMCs were exposed to both *Leishmania* species for 7 days, enhanced splicing of IL-32γ mRNA occurred with an increase in IL-32α and IL-32β isoforms expression, resulting in a high amount of IL-32 in the cells. The induction of both IL-32 mRNA and protein reveals to be dependent on several PRRs, namely NOD2, TLR4 and Dectin-1. We showed that genetic variants of the *IL32* gene influenced IL-32γ mRNA expression. Moreover, we could demonstrate that these investigated genetic variations influenced the production of innate and acquired *Leishmania*-induced cytokine production by primary human PBMCs. Of high interest, our data suggest that the *IL32* SNP (rs4786370) is associated with susceptibility to ATL. In addition, the *IL32 SNP* (rs4349147) was associated with the clinical outcome of ATL patients, being more frequent among ML than LCL patients. Lastly, here we demonstrated that the IL-32γ mRNA expression in lesions of ATL patients was positively correlated with the expression of several proinflammatory cytokines, such as *IL1A*, *IFNG*, *IL17*, and *IL22*. In addition, IL-32α mRNA expression was positively correlated with *IL1A* and *IL17* mRNA expression whereas IL-32β mRNA expression was associated with *IL17* and *IL22* mRNA expression.

In the present study, it was shown that promastigote antigens are able to induce early expression of IL-32γ in human PBMCs (Figs A and B). In contrast, after 7 days of exposure to both *Leishmania* species, human PBMCs predominantly expressed IL-32α and IL-32β mRNA (Fig 1C and 1D). In agreement with our findings using promastigotes antigens, previously studies have shown that the infection of THP-1 macrophages with live promastigote forms of *L*. *amazonensis* or *L*. *braziliensis* as well as the adittion of amastigotes forms in PBMCs lead to the early induction of IL-32γ mRNA expression [35,36]. In addition, promastigote antigens from *L*. *amazonensis* were able to induce higher levels of IL-32γ than *L*. *braziliensis* in PBMCs, confirming the previous results obtained with THP-1 macrophages [36]. In the present study, we have shown that the expression of IL-32 isoforms is dependent on duration of exposure to *Leishmania* antigens, which might reflect the ability of the parasite to modulate IL-32γ splicing as the infection progress. Moreover, the subset of cells studied, myeloid (24 h) or lymphoid (7 days), may influence the expression of the different IL-32 isoforms. Interestingly, the highest concentrations of intracellular IL-32 protein were seen after 7 days of exposure to *Leishmania* spp. (Fig 1F). Of note, since IL-32 isoforms have different biological properties in regard to the induction of inflammation [46], to determine which IL-32 isoform is being induced for *Leishmania* spp. taking in account the subset of cells and the different stages of disease (early or chronic) might be relevant to understand its relation with the immunopathogenesis of the disease.

Although it was reported previously that *Leishmania* spp. can induced IL-32 in human cells and it is positively associated with cytokine production *in vitro* and *in vivo* [35,36], which receptor is involved in this process is unknown. Using a functional genomics approach, we were able to identify the receptors that are important for *Leishmania s*pp. mediated induction of IL-32γ, the most inflammatory isoform of IL-32. Functional genomics revealed to be a powerful approach to identify novel pathways in infectious diseases, such as candidemia, tuberculosis and Lyme disease [47,48]. Of high interest, using a genomics approach and functional validation, we could show that NOD2, TLR4 and Dectin-1 receptors are involved in *Leishmania*-induced IL-32γ expression. Several C-type lectins, such as MR and Mincle have been described as recognition receptors for *Leishmania* [49–51] [12,52]. It is unlikely that MR is involved in the induction of IL-32γ since there were no SNPs identified that modulated the IL-32γ expression (S1B Fig).

Previous data have shown the relevance of IL-32γ for cytokine and microbicidal molecules production during *Leishmania* infections using THP-1 macrophages [36]. In order to demonstrate the role of IL-32 in *Leishmania*-induced cytokine production by primary cells, we described genetic variations within *IL32* that alter IL-32γ mRNA expression and proinflammatory cytokines produced by cells from innate and acquired immunity; thus, we have shown that depending on the *Leishmania* spp., genetic variations in *IL32* lead to functional consequences modulating IL-32γ expression and the production of proinflammatory cytokines in human primary cells (Figs 3,4 and 5). As all cytokines evaluated in this study have been shown to be involved in immunopathogenesis of leishmaniasis [15,16,53] and in the parasite control [54,55], the present data indicate a pivotal role of IL-32 in the induction of cytokine production after *Leishmania* spp. exposure. The results presented here confirm that Il-32γ is a crucial cytokine to control cytokine production induced by *Leishmania* spp. in macrophage cell lines as well as in primary cells.

To investigate whether these genetic variations in *IL32* could be associated with susceptibility or resistance to ATL, we evaluated the differences in genotypes frequencies of ATL patients compared to controls. Genetic variations have been described associated with suscepitibility to complicated skin and skin structure infections (cSSSIs) and HIV infections [45,56]. Likewise in our study, we observed an increased frequency of *IL32* rs4786370-CC genotype in controls. This genotype was linked with high IL-32γ and IL-22 induction in PBMCs after exposure to *L*. *amazonensis* antigens, which suggest a possible role of IL-32 in decreasing the risk of developing ATL. Despite of *in vitro* cytokine alterations, the other *IL32* genetic variants (rs4349147; rs1555001) presented similar frequencies between the groups of ATL patients and controls what could be ascribed due the low number of individuals included in the study. In addition to the low number, other relevant aspect for genetic studies is the presence of a heterogeneity population present in Brazil. Using ancestry-informative DNA polymorphisms in individuals from the four most populous regions of the country, a previous study has unraveled great ancestral diversity between and within different regions of the country [57]. Thus, although the sex-related differences in ATL susceptibility was not addressed in the present study, data from across Brazil show that males are more susceptible to develop cutaneous manifestations of leishmaniasis than females [58]. Since this is first study investigating the role of genetic variations in *IL32* associated with susceptibility to ATL, the use of a collective dataset including for genetic associated studies with large number of samples as well as matched asymptomatic and healthy controls, epidemiological variables and host biological factors respresent a promising new endeavor within the scientific community in the field of *Leishmania* research in Brazilian subjects.

After stratifying patients for their *IL32* genotype and correlate it with clinical manifestation of the disease, it was observed the allele G of the *IL-32* rs4349147 was more frequent in patients with LCL than ML. The G allele was associated with increased levels of IL-32γ, IL-6, IL-1β and IL-22 in PBMCs of healthy individuals exposed to *L*. *amazonensis* antigens, and with IL-1β and IL-22 to *L*. *braziliensis* antigens. Thus, these findings suggest that allele G might contribute to the development of the less severe form of the disease conferring protection against ML. Future studies exploring whether the *IL-32* rs4349147 is associated with the production of immune mediators are worthwhile in a cohort of ATL patients.

Based on the expression of individual genes coding for immune host defense and being described related with IL-32 expression [29], early and late lesions from *L*. *braziliensis* skin that are transcriptionally indistinguishable [42] could be clearly separated from normal skin based on mRNA levels. Expression of *IL32* and other genes that promote severe inflammation including cytokines, chemokines and also metalloproteinases, which can degrade the extracellular matrix leading to more tissue damage, were higher in *L*. *braziliensis* lesions. Positive correlations between IL-32 and IL-22 were observed in *L*. *braziliensis* lesions. IL-22, as well as IL-32, have been described to be important for host defense against microorganisms and antimicrobial peptide secretion [28,29,59].

To investigate the associations between Il-32 isoforms and other proinflammatory cytokines *in vivo*, we evaluated the expression of *IL1A*, *IL1B*, *IFNγ*, *IL17*, and *IL22* in lesions of ATL patients and studied their correlations with Il-32 alpha, beta and gamma. The mRNA of the three IL-32 isoforms, being IL-32γ > IL-32β > IL-32α were detected in the lesions of ATL patients. In the lesions of of ATL patients, IL-32γ mRNA expression was correlated with *IL1A*, *IFNγ*, *IL17* and *IL22*. Although IL-32γ is the most prevalent isoform in the lesions of ATL patients (present data; [35]), we also evaluated whether IL-32β and IL-32α isoforms were associated with proinflammatory cytokines in ATL lesions. IL-32β mRNA was correlated with and *IL17* and *IL22*; IL-32α with *IL1A* and *IL17* (S1, S2 and S3 Figs). These results suggests that the IL-32γ isoform can be associated with a mixed Th1/Th17 profile whereas IL-32β and IL-32α can be more associated with a Th17 profile. It is known that IL-32γ can induce a mixed Th1/Th17 acquired immune response [33,60]. Previous data has shown that in other inflammatory skin diseases such as Psoriasis and Hydradinitis Superativa, IL-32β was the main isotype that was expressed [61]. Although IL-32β is known to present proinflammatory properties [43], it has been shown that IL-32β induces IL-10 production [62]. IL-32α is regarded as anti-inflammatory member of the IL-32 family which is able to inhibit NK cell activation and JAK/STAT pathway [63]. Thus, in our study all three isoforms are associated with proinflammatory cytokines in ATL, it is possible that IL-32β and IL-32α act as anti-inflammatory mediators in the lesions, being important to decrease the inflammatory process, however, whether IL-32β or IL-32α acts as an anti-inflammatory mediators in lesions of ATL patients is not known at this moment.

In the present study we demonstrated that IL-32γ mRNA expression in the lesions of *Leishmania*-infected patients strongly correlated with cytokines related to skin inflammation, such as IL-17, IL-22 and IL-1α. Of high interest, IL-22 was found to be highly correlated with IL-32γ expression. IL-22 is known as crucial mediator in cutaneous host defense against extracellular microorganisms, however, it seems not relevant to protect against intracellular micoorganisms [64]. In addition, IL-22 presents proinflammatory and anti-inflammatory properties, promoting wound healing/repair by activationg epithelial cells and fibroblasts [64,65]. In *L*. *major-* or *L*. *braziliensis*-infected mice the production of IL-22 decreases the lesion size without any effect on parasite load, indicating that IL-22 is more associated with the control of inflammation and wound healing than with parasite control in cutaneous leishmaniasis [23,66]. The role of IL-17 in ATL has been studied. Several studies suggest that IL-17 can be pathogenic in ML patients [16,20] whereas other suggests a protective role for IL-17 in LCL [67]. In regard to IL-1 family, it has been shown that IL-1α and IL-1β are expressed in ATL lesions caused by *L*. *braziliensis*. The expression of IL-1β in the lesions of *L*. *braziliensis*-infected LCL patients has been associated with immunopathology [53]. In opposite to humans, in mouse models, IL-1β has been shown important for protection in C57BL/6 mice infected with *L*. *braziliensis* or *L*. *amazonensis* [55]. Thus, the exact mechanism in how IL-32 isoforms modulates the production of proinflammatory cytokines and whether these connections contributes to immunopathology or wound healing remains to be investigated in leishmaniasis.

In conclusion, this study raises the concept of an important role for endogenous IL-32 in modulation of the inflammatory response in humans that influences susceptibility to and progression of localized cutaneous and mucosal leishmaniasis. In ATL, IL-32 is a novel target and may have promising therapeutic potential in the future. However, several aspects of the IL-32 biological function of IL-32 in human immune cells remain elusive. The precise molecular mechanisms mediated by IL-32 in lesions of *Leishmania* patients, including inflammatory pathways and control of parasite load, needs to be elucidated. Moreover, future studies should reveal whether the functional effects of genetic variations in *IL32* observed in human PBMCs are also exerted in the ATL lesions environment.

## Supporting information

S1 Table TaqMan SNP assays. (DOCX)

S1 Fig PBMCs from healthy donors from the 200FG cohort were stimulated with lysates (50 μg/ mL) of *L. amazonenis* and *L. braziliensis* for 24 hours. (A) IL-32γ mRNA expression in PBMCs isolated from individuals carrying a frame shift mutation in the NOD2 receptor (rs2066847- leu1007insC n = 3) compared to individuals carrying no mutation (Wt n = 20). The individuals were stratified according with different genotypes of (A) *TLR4* rs4986791 (AA n = 26; AG n = 4), *TLR2* rs5743708 (GG n = 32; GA n = 2), (B) *CLEC7A* rs16910526 (AA n = 32; AC n = 1; CC n = 1), *CD206* rs1926736 (AA n = 5; AG n = 20; GG n = 18), and IL-32γ gene expression was determined. The data shown are the mean ± SEM of fold change in IL-32γ expression induced by *Leishmania* spp. normalized to RPMI control.
(TIFF)

S2 Fig Correlation of IL-32β with *IL1B, IL1A, IFNG, IL17* and *IL22* gene expression in skin biopsies of ATL patients. The data shown are log2 of Relative expression adjusted for beta-2 microglobulin expression (n = 56).
(TIFF)

S3 Fig Correlation of IL-32α with *IL1B, IL1A, IFNG, IL17* and *IL22* gene expression in skin biopsies of ATL patients. The data shown are log2 of Relative expression adjusted for beta-2 microglobulin expression (n = 56).
(TIFF)
