# Supplementary material for: Genetic variation in Interleukin-32 influence the immune response against New World Leishmania species and susceptibility to American Tegumentary Leishmaniasis
Source: PLoS Negl Trop Dis. 2020 Feb 5;14(2):e0008029. doi: 10.1371/journal.pntd.0008029 (PMC7028298; doi:10.1371/journal.pntd.0008029)
Supplement: S1 Table — (DOCX) [file pntd.0008029.s001.docx]

**S1 Table. TaqMan SNP assays**

| **Gene** | **SNP ID** | **Region** | **Nucleotide Change** | **TaqMan Assay ID** |
| --- | --- | --- | --- | --- |
| *IL-32* | rs4786370 | Promoter | T >C | C__27972515_10 |
| *IL-32* | rs4349147 | Enhancer | G >A | C__32104871_10 |
| *IL-32* | rs1555001 | Intron | T >A | C_11186529_10 |

^a^The first nucleotide is the ancestral nucleotide.
