# Supplementary figures and images for: Genetic variation in Interleukin-32 influence the immune response against New World Leishmania species and susceptibility to American Tegumentary Leishmaniasis
Source: PLoS Negl Trop Dis. 2020 Feb 5;14(2):e0008029. doi: 10.1371/journal.pntd.0008029 (PMC7028298; doi:10.1371/journal.pntd.0008029)

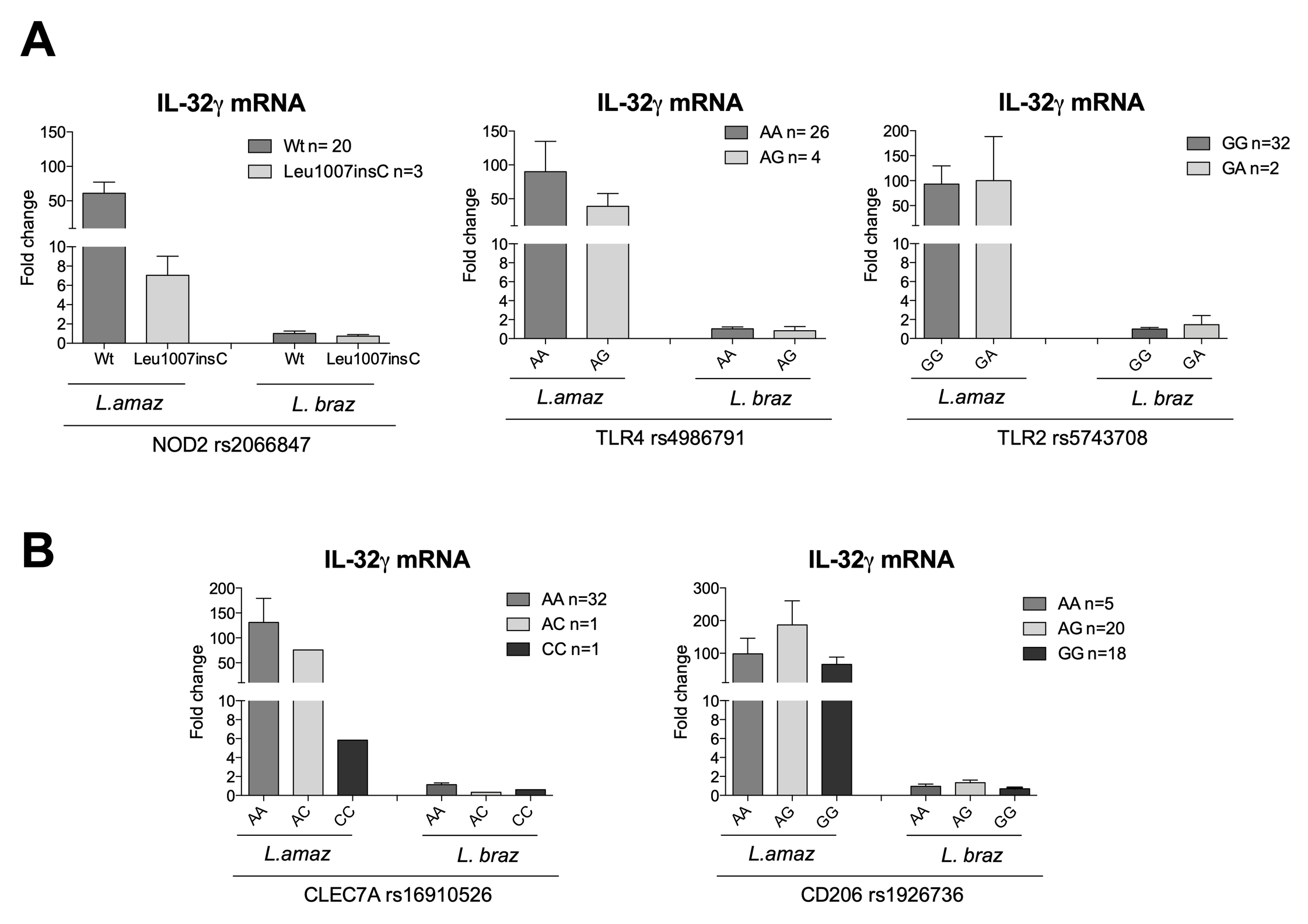

Supplement: S1 Fig — (A) IL-32γ mRNA expression in PBMCs isolated from individuals carrying a frame shift mutation in the NOD2 receptor (rs2066847- leu1007insC n = 3) compared to individuals carrying no mutation (Wt n = 20). The individuals were stratified according with different genotypes of (A) TLR4 rs4986791 (AA n = 26; AG n = 4), TLR2 rs5743708 (GG n = 32; GA n = 2), (B) CLEC7A rs16910526 (AA n = 32; AC n = 1; CC n = 1), CD206 rs1926736 (AA n = 5; AG n = 20; GG n = 18), and IL-32γ gene expression was determined. The data shown are the mean ± SEM of fold change in IL-32γ expression induced by Leishmania spp. normalized to RPMI control. (TIFF) [file pntd.0008029.s002.tiff]

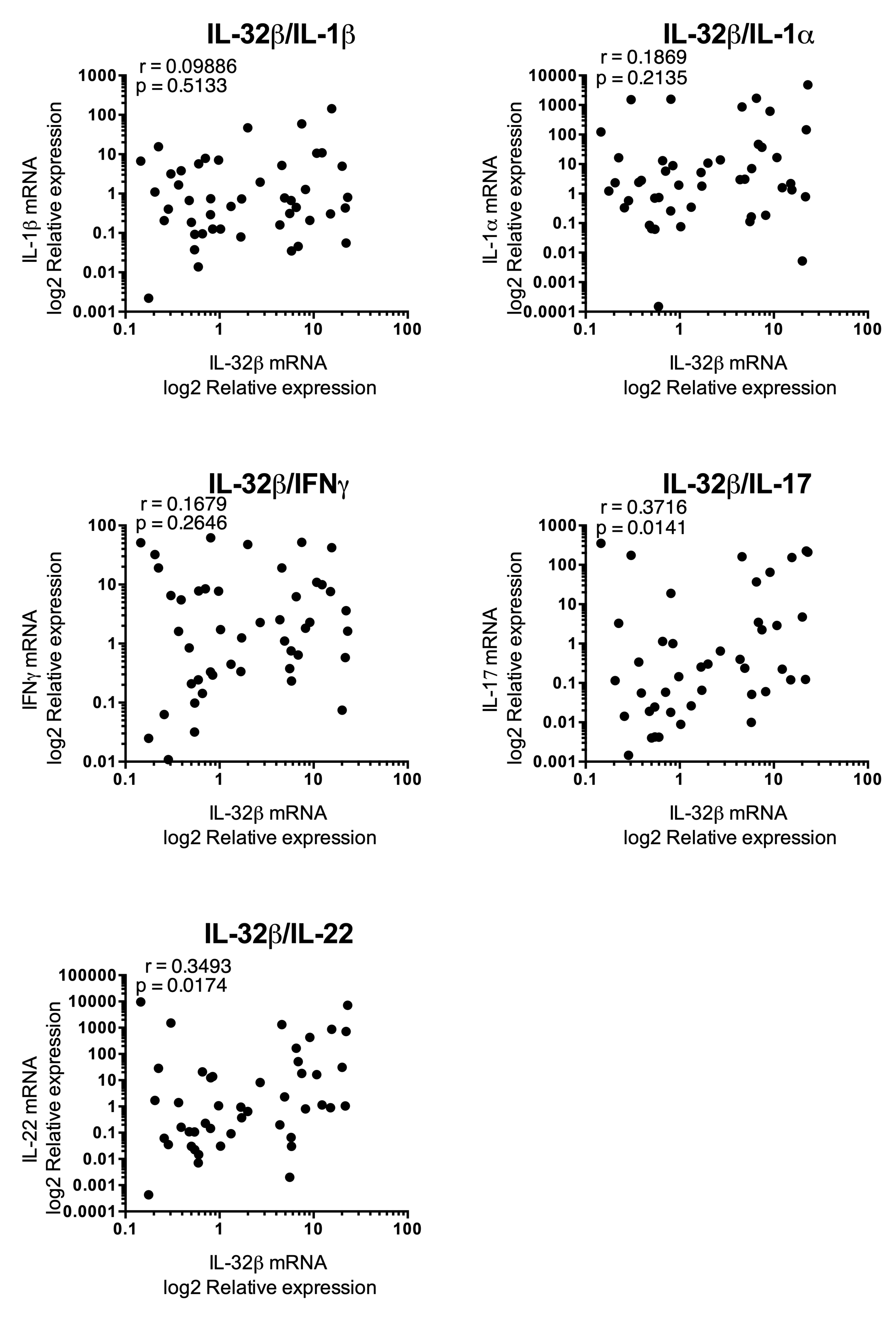

Supplement: S2 Fig — The data shown are log2 of Relative expression adjusted for beta-2 microglobulin expression (n = 56). (TIFF) [file pntd.0008029.s003.tiff]

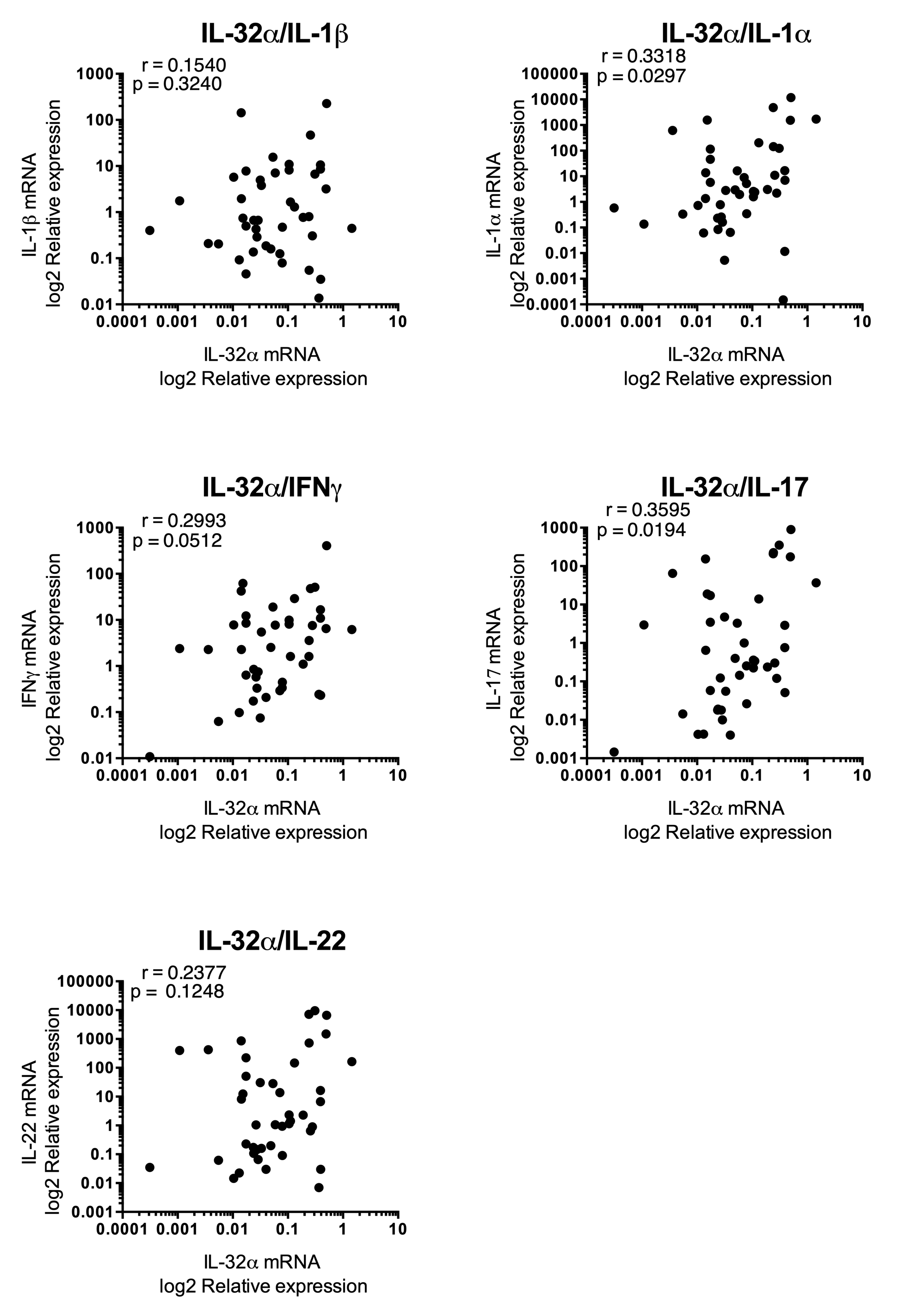

Supplement: S3 Fig — The data shown are log2 of Relative expression adjusted for beta-2 microglobulin expression (n = 56). (TIFF) [file pntd.0008029.s004.tiff]
